# Chronic Subdural Hematoma Associated with Congenital Arachnoid Cysts: Management Dilemmas

**DOI:** 10.7759/cureus.2550

**Published:** 2018-04-30

**Authors:** Sashanka Kode, Ajay Hegde, Girish Menon

**Affiliations:** 1 Neurosurgery, Kasturba Medical College, Manipal 576104, Karnataka, India.

**Keywords:** subdural drainage, arachnoid cyst, subdural haemorrhage

## Abstract

Chronic subdural hematoma (CSDH) is one of the commonest diseases encountered by a neurosurgeon in daily practice. It is however rarely seen in young patients. Congenital arachnoid cysts have been implicated in both traumatic and spontaneous CSDH in young individuals. Optimum treatment strategies to address the CSDH and arachnoid cyst are not very well described. We report a young gentleman who was treated for a CSDH with arachnoid cysts, two months after a mild head injury. The patient was operated with a simple burr hole drainage of hematoma with a drain. He was discharged with no further need to address the arachnoid cyst. CSDH associated with arachnoid cysts can be treated with simple burr hole drainage. Craniotomy, fenestration and cerebrospinal fluid (CSF) diversion should be reserved only as secondary procedures.

## Introduction

Chronic subdural hematoma (CSDH) is commonly seen in the elderly and is often attributed to minor inertial brain injury resulting in bridging vein injuries [1]. The congenital arachnoid cyst has been established to be a statistically significant risk factor for CSDH after mild head injury in young patients [2]. Chronic subdural hematomas associated with arachnoid cyst pose issues in terms of diagnosis and optimal management strategy. We report a case of a young patient with a Sylvian arachnoid cyst and CSDH and highlight the management dilemmas.

## Case presentation

**Figure 1:**
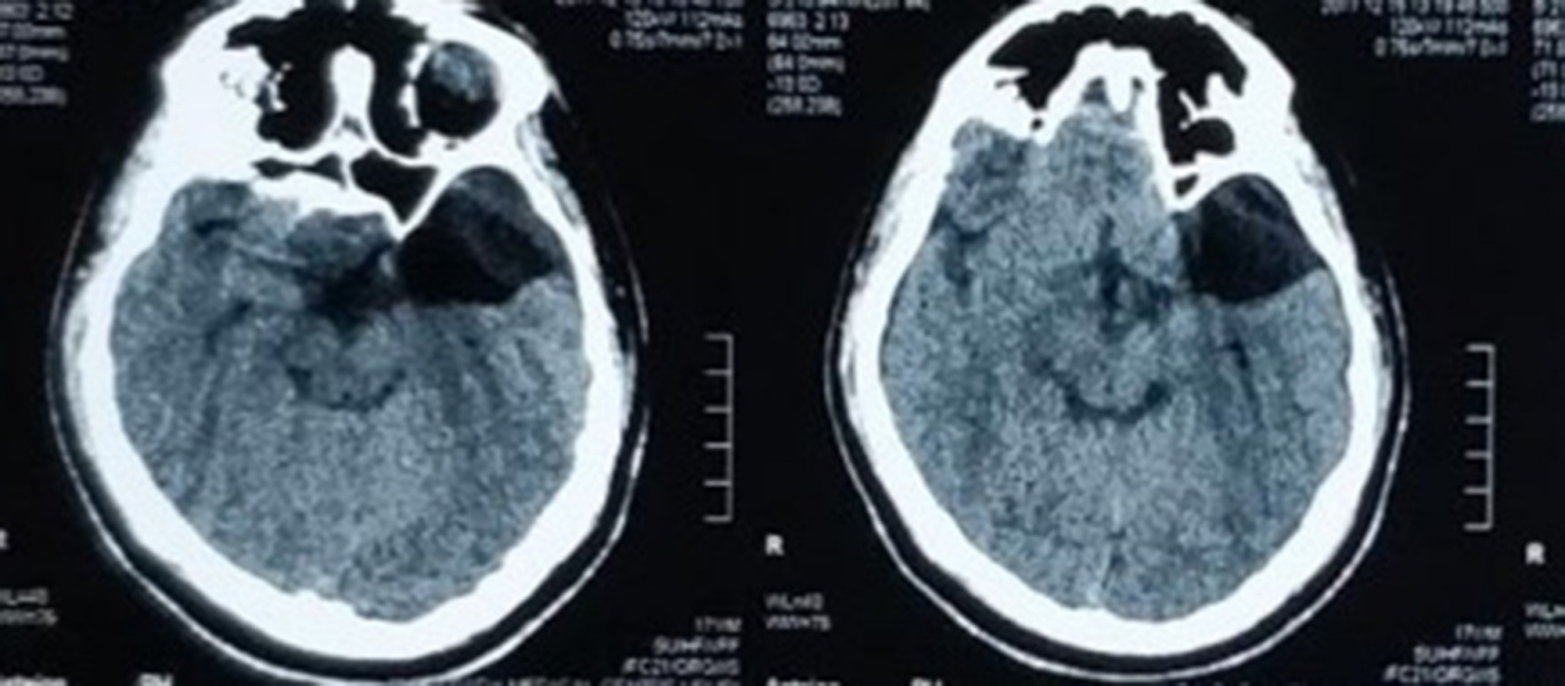
Left temporal pole arachnoid cyst extending into the left sylvian fissure.

A 21-year-old male had presented to the emergency with the history of one episode of generalized tonic-clonic seizures followed by altered sensorium and repeated episodes of vomiting for one day. His parents also noticed a paucity of limb movements on the right side. Past history revealed that he had sustained a minor head injury following a trivial road traffic accident two months ago, following which he was stable except for an occasional headache. A computed tomography (CT) scan brain was done two weeks after the initial trauma which revealed an incidental left Sylvian Type II arachnoid cyst (Figure 1).

**Figure 2:**
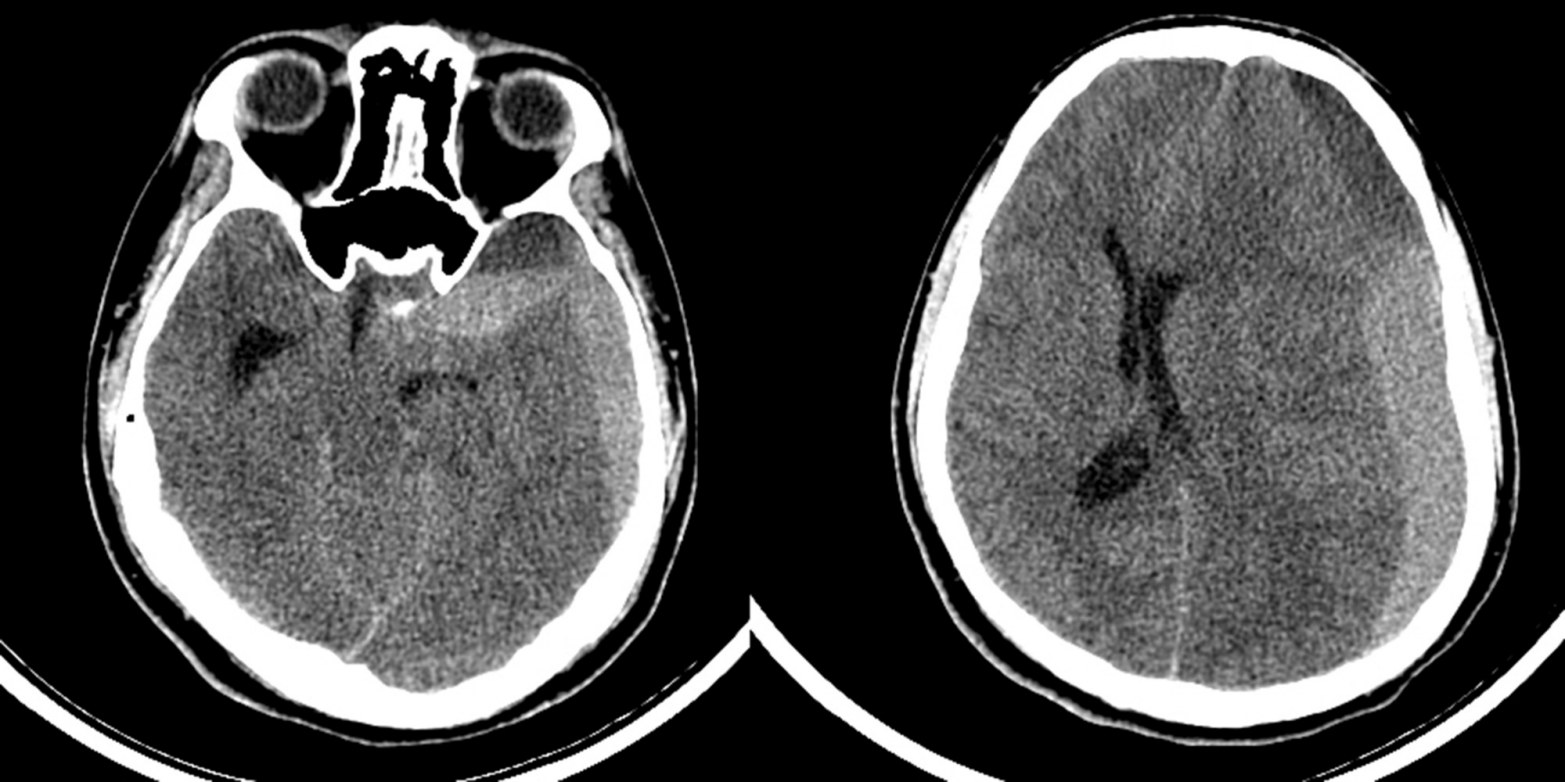
Left acute on chronic subdural hemorrhage with blood in the arachnoid cyst and midline shift to right.

He was kept on observation with symptomatic treatment for his headache until the time he presented with seizures and altered sensorium. On admission, his Glasgow coma scale (GCS) was seven (E1V1M5). His pulse rate was 56 bpm and he had features of left uncal herniation. An emergency CT of the brain was done which revealed an acute on CSDH on the left side with bleed into the cyst cavity and a significant midline shift (Figure 2).

**Figure 3:**
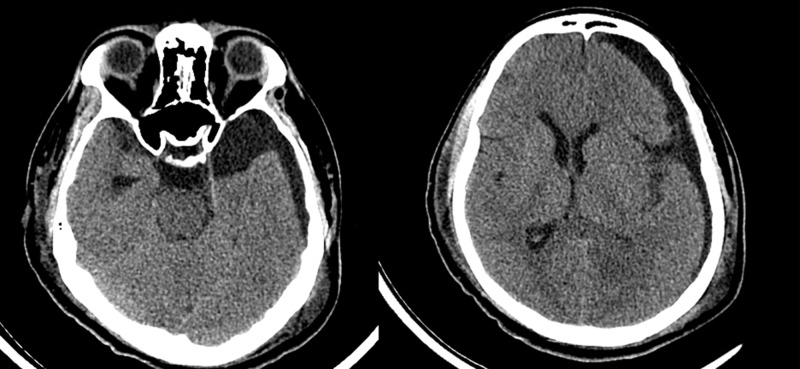
Post-operative scan with evacuation of hematoma and resolution of midline shift.

Emergency surgery was performed and dark machine oil coloured fluid was evacuated through two burr holes. He showed good improvement neurologically post evacuation with complete hematoma evacuation and significant improvement of midline shift on follow up scan (Figure 3). His post-operative period was complicated with cerebrospinal fluid leak from the parietal burr hole on suture removal followed by a pulsatile swelling in the frontal burr hole, which was managed conservatively with acetazolamide and pressure dressing of the wound. The swelling spontaneously disappeared in a week and the patient has been asymptomatic since then.

## Discussion

The association of arachnoid cysts with CSDH seems to be more than coincidental. In a large retrospective study of 48,417 patients arachnoid cysts were identified in 661 patients (1.4%) [3]. Parsch et al., analyzing 658 patients with CSDH, observed arachnoid cysts of the middle cranial fossa in 2.43% of patients suggesting a fivefold greater prevalence of arachnoid cysts in patients with CSDH [4]. A similar observation was made by Mori et al. in their study done of 541 cases of surgically treated CSDHs of whom 12 cases had arachnoid cyst (2.2%) [2]. Wu et al. have systematically analyzed and summarized all the 182 cases reported in the literature so far including 14 of their own series and conclusively state that arachnoid cyst is an important risk factor for the development of subdural hemorrhage [5]. The exact mechanism of development of SDH in patients with arachnoid cyst is unclear. Page et al. have proposed two theories to explain this phenomenon. The first hypothesis is that in response to mild trauma, flow changes within the cerebrospinal fluid (CSF) could be magnified by the arachnoid cyst leading to rupture of bridging veins or vessels in the cyst wall. The second assumption is that arachnoid cyst is less compliant than normal brain resulting in reduced intra-calvarial cushioning following minor trauma. Thus, hemorrhage may occur from bridging veins ipsilateral or contralaterally resulting in subdural hematomas [6].

In addition, De Oliveira et al. [7] have suggested a rare association between aneurysm and arachnoid cyst and there have also been multiple case reports where an aneurysm rupture into the arachnoid cyst presenting as a subdural haemorrhage [8,9]. Literature suggests that paediatric, juvenile, and young adult patients with intracranial arachnoid cyst are at the greatest risk of complicated CSDH. Similarly, a male preponderance has also been noted in most series. A temporal or middle cranial fossa location, larger cyst size and history of head injury seem to be other risk factors for the occurrence of a CSDH. Our patient satisfies all the above criteria being a young male with a large temporal arachnoid cyst.

The optimal treatment strategy is unclear. Burr hole drainage of the clot without manipulation of the cyst, craniotomy/craniectomy with drainage of the clot and excision/fenestration of the cyst have all yielded similar results. Summary of all the published 169 cases by Wu et al. [6] suggests that of the 85 patients who underwent initial burr hole drainage, seven (8.2%) experienced early recurrence of CSDH. Of the 66 patients underwent craniotomy or craniectomy with/without arachnoid cyst resection or fenestration, only one (1.5%) experienced early recurrence of CSDH. Wu et al. thus suggest that burr hole drainage is the first-choice surgical procedure in symptomatic patients and is still effective in some recurrent cases. Fenestration or resection of the arachnoid cyst membrane is not a requisite in patients with previous asymptomatic arachnoid cysts. However, large numbers are required to evolve a standard treatment protocol. Our patient did extremely well with simple burr hole drainage of the subdural clots.

## Conclusions

Young patients with arachnoid cyst are at a high risk of developing a chronic subdural hematoma following trivial head trauma and should be kept under close observation. Burr hole drainage of the hemorrhage provides a good clinical outcome. The need for surgery for the arachnoid cyst is still unclear. The ideal management would be a burr hole drainage of hematoma. In the case of recurrence reoperation by burr hole drainage may still provide satisfactory results in the majority of cases. Craniotomy should be reserved only for cases which recur after resurgery. Cyst fenestration or excision is rarely required and has not been proved to be more efficacious than evacuation of hematoma in altering the ultimate outcome.
